# The Management of Dissolved Oxygen by a Polypropylene Hollow Fiber Membrane Contactor Affects Wine Aging

**DOI:** 10.3390/molecules26123593

**Published:** 2021-06-11

**Authors:** Francesco Errichiello, Luigi Picariello, Antonio Guerriero, Luigi Moio, Martino Forino, Angelita Gambuti

**Affiliations:** Department of Agricultural Sciences, Section of Vine and Wine Sciences, University of Naples ‘Federico II’, Viale Italia, 83100 Avellino, Italy; francesco.errichiello@unina.it (F.E.); luigi.picariello@unina.it (L.P.); a.guerriero1385@gmail.com (A.G.); luigi.moio@unina.it (L.M.); forino@unina.it (M.F.)

**Keywords:** oxidation, membrane contactor, wine, polymeric pigments

## Abstract

Background: Numerous oenological practices can cause an excess of dissolved oxygen in wine, thus determining sensory and chromatic defects in the short- to long-term. Hence, it is necessary to manage the excess of oxygen before bottling. Methods: In this study, the management of the dissolved oxygen content by a polypropylene hollow fiber membrane contactor apparatus was performed in two wines from different grape varieties (Aglianico and Falanghina). The wines were analyzed after an 11-month aging. Anthocyanins and acetaldehyde content were evaluated by HPLC. In addition, other phenolic compounds and chromatic characteristics were analyzed by spectrophotometric methods. NMR and HR ESIMS analyses were conducted to evaluate the amount of pyranoanthocyanins and polymeric pigments. Results: After 11 months of aging, in both wines a decrease of free and total SO_2_ with respect to initial values was detected. In the wines with the highest dissolved oxygen levels, a more remarkable loss was observed. No significant differences in terms of color parameters were detected. In red wine with the highest oxygen content, a massive formation of polymeric pigments and BSA reactive tannins was observed, as opposed to wines with lower oxygen levels. Conclusion: The study demonstrated that the membrane contactor can prove a successful tool to manage dissolved oxygen in wines as to prevent their oxidative spoilage.

## 1. Introduction

Wine is a chemically dynamic system, and even after fermentation its composition continues to evolve during the storage. These post-fermentation changes are referred to as aging, but a distinction is to be made between the changes occurring during the maturation phase (e.g., bulk storage of wine in tank or barrel), when the winemaker’s intervention is still allowed, and those taking place during the aging phase once wine has been sealed in bottles and the only possible intervention is limited to the selection of the most appropriate storage conditions.

Among wine compounds, phenolics are those mostly affected by aging. They originate from grapes (flavonoids and non-flavonoids) and constitute one of the most important wine quality parameters. During winemaking and aging, phenolics mainly undergo oxidation reactions, which not only affect the phenolic composition itself but also determine changes in terms of sensory characteristics, such as color and astringency.

Phenolic compounds are the primary reactants to be oxidized in presence of oxygen and metals (Fe^3+^_,_ Cu^2+^), giving rise to a cascade of chemical transformations that may result in an excessive deterioration of wine [1]. Wine oxidation consists of a series of reactions: first, oxygen is reduced to hydrogen peroxide by interacting with transition metals, including iron and copper ions, in the presence of catechol subunits that are oxidized to quinones [2]. Quinones strongly react with nucleophilic compounds, such as antioxidants (sulfur dioxide, glutathione, ascorbic acid), desirable aroma volatile thiols (i.e., 3-sulfanylhexanol), undesirable aroma thiols (i.e., hydrogen sulfide), amino acids (i.e., phenylalanine, methionine) and numerous polyphenols (mainly flavanols). The products of these reactions may lead to the formation of condensed polymeric pigments—particularly important in red wines—or even to the loss of color and varietal characters [3]. In a subsequent step, ferrous or cuprous species react with hydrogen peroxide by the Fenton reaction to yield the hydroxyl radical, a strong oxidant, capable of reacting with all organic constituents in proportion to concentration [4]. The most abundant organic compound in wine is ethanol, which is converted into acetaldehyde once oxidized by the hydroxyl radical.

As a consequence of oxidation, in red wine native anthocyanin pigments are quickly transformed into more stable pigments via various types of reactions such as aldehyde-mediated condensation reactions with tannins and cyclo-addition reactions leading to the formation of pyranoanthocyanins [5]. Oxidation reactions also contribute to modifying the wine astringency by changing the tannin structure as a consequence of intra and inter molecular reactions mediated by oxygen [6]. These “stabilized products” anthocyanin or pigmented tannins persist much longer in wine than their initial forms [7]. Thus, low amounts of oxygen in red wine are important to stabilize either color or astringency. Pasteur himself, in his studies on wine, theorized that only when a wine is exposed to oxygen can it develop attributes that make it a finely aged high-quality product. During winemaking, oxygen plays a crucial role in the fermentation process. It promotes the yeast biomass synthesis and favors a sound fermentation. Several studies have shown that the risk of stuck and sluggish fermentations is reduced after oxygen additions of 10–20 mg/L [8].

In white wines oxidation is usually associated with important changes in color. A brown color is normally unwanted, because it is a sign of oxidation in table white wine. Brown coloration can be induced by enzymatic or chemical oxidation mediated by oxygen. The latter is slower than the enzymatic-induced oxidation. White wine is generally more sensible to O_2_ than red wine. Even small additions of O_2_ to white wine can lead to loss of aroma, especially fruitiness with the appearance of off-flavors described as caramel, rancid, farmed-feed, honey-like and cooked vegetables. The quinones generated from oxidation can react with thiols by the Michael addition reaction or generate H_2_O_2_, as reported above. The oxidative environment through all the phases of winemaking is positively correlated to the formation of these products. During the aging of red wine in oak barrel, the oxidative process also induces the formation of sotolone through the oxidation of threonine or by the reaction of acetaldehyde with α-ketobutyric acid. The oxidative degradation of phenylalanine and β-phenylethanol in a barrel also leads to higher concentrations of phenylacetaldehyde [9].

As described above, oxygen can have either beneficial or detrimental effects on the wine quality. The level of oxygen exposure of wine during winemaking or aging is crucial as it can affect the final product. Singleton [10] estimated the amount of oxygen that a white or a red wine could absorb before oxidative defects emerge. In white wine, the toleration is around 10 air saturations as opposed to red wine, which can tolerate more than 30 air saturations (180 mL O_2_/L). He also recommended about 10 saturations to improve the quality of red wine.

Oxygen management during the phases of vinification and storage is therefore important and must be handled according to the knowledge acquired as to avoid the insurgence of oxidative characters. After the winemaking, wine usually undergoes a series of stabilization practices such as decanting, refrigeration and filtration that can determine an uncontrolled oxygen inlet. Additionally, new vinification methods that use stainless steel tanks and systems allowing a controlled oxygen micro-supply (micro-oxygenation) are now common. Even when specific efforts have been made to produce wines, which are as resistant as possible to further oxygen intakes, all the uncontrolled dissolved oxygen in wine can determine a further development of oxidize characters once bottled. In this context, the use of a membrane contactor to manage oxygen in wine before bottling might be a successful strategy in order to obtain the best possible wines.

Membrane contactors are among the most used industrial systems, and this technology has been proven useful in a range of liquid/liquid and gas/liquid applications in fermentation, pharmaceuticals, wastewater treatment, chiral separations, semiconductor manufacturing, carbonation of beverages, metal ion extraction, protein extraction, VOC removal from waste gas, osmotic distillation and wine dealcoholization [11,12]. It is a device that achieves gas/liquid or liquid/liquid mass transfer without dispersion of one phase within another. Although membrane contactor technology was introduced as a tool for gas management in wines [13,14], until now few studies have dealt with the effectiveness of its application before bottling to regulate the evolution of white and red wine during bottle aging.

In this study, a partial deoxygenation was performed on two monovarietal wines: Aglianico and Falanghina. The effect of oxygen removal on several wine parameters as free and bound SO_2_ and acetaldehyde content was evaluated after 11 months of bottle aging. For red wine, the effect on chromatic characteristics and main phenolic compounds was also evaluated.

## 2. Results and Discussion

In this study two commercial wines (Aglianico (R) and Falanghina (W)) were submitted to a deoxygenation process by using membrane contactor technology to obtain three wines with decreasing levels of dissolved oxygen (high (H), medium (M) and low (L) for each wine) before the bottling phase. After 11 months of bottle aging, the effects on sulfur dioxide, acetaldehyde, chromatic characters, polymeric pigments, VRF, BSA-tannins and total phenolics were evaluated.

### 2.1. Sulfur Dioxide

The concentration of free and total SO_2_ was monitored after 11 months of aging in the Aglianico wine (Table 1). For all samples, a loss of SO_2_ with respect to bottling time (free SO_2_ 18 mg/L, tot SO_2_ 43 mg/L) was observed and the greatest decline in the values of total SO_2_ was observed in the wines with higher oxygen contents at bottling (RHO_2_ and RMO_2_), as expected.

During the aging process, the most abundant forms of free SO_2_ at the pH of the wine, the bisulfite ion (HSO_3_^-^ in equilibrium with molecular SO_2_), is consumed by the reaction with hydrogen peroxide and several electrophilic wine components, such as those produced by the oxidation cascade, including quinone and acetaldehyde [15]. In winery settings, it is common practice measuring the so-called “free SO_2_” that is the sum of molecular SO_2_ and bisulfite ion. This latter compound can form covalent adducts with electrophiles, called SO_2_ binders, that can be classified as weak or strong based on the dissociation constant of the sulfite adducts formed. These sulfite-adducts are called bound SO_2_ and the sum of free and bound SO_2_ gives the “total SO_2_”. The free and bound SO_2_ are in equilibrium with each other in wine. Moreover, during oxidation, by the equilibrium between these two forms of SO_2_ and by the consumption of free SO_2_, the combined form is released to restore the equilibrium. In all treated red wines, values of free SO_2_ below 3.84 mg/L were detected after 11 months of bottle aging. These values are well below the quantification limit of official methods of analysis of free SO_2_ [16]; therefore, it is more correct to consider negligeable the value of free SO_2_ while the values of total SO_2_ were lower in samples bottled with higher content of dissolved oxygen (RHO_2_). The shift between free and combined during wine aging might be the reason why decreasing levels of total SO_2_ were detected in wines as dissolved oxygen at bottling increased. Apart from the consumption of SO_2_ owing to oxidation reactions, part of it can be also lost during the aging due to the reactions of sulfur dioxide with flavanols. The mechanism of formation of 4*ß*-sulfonated products is still uncertain. It is hypothesized that monomeric 4*ß*-sulfonated derivatives are formed by the acid-catalyzed depolymerization of proanthocyanidins [17].

The concentrations of free and total SO_2_ (Table 1) were monitored after 11 months of aging in white wine whose behavior turned out to be the same as that in red wine. Further, in this case a loss of free and total SO_2_ with respect to the initial values was detected (free SO_2_ 26 mg/L and tot. SO_2_ 89 mg/L). In addition, wines with higher content of oxygen at bottling (WHO_2_) showed a lower content of free and total SO_2_.

Acetaldehyde, formed by the metal-catalyzed oxidation of ethanol during wine oxidation, was higher at 11 months of aging in sample WHO_2_ with respect to sample WMO_2_, and WLO_2_ as expected given the lower content of free and total SO_2_ in WHO_2_ samples (Table 2). Because the weight ratio between acetaldehyde and sulfur dioxide is 1.4/1 (1.4 mg of SO_2_ consumed per 1 mg of CH_3_CHO), we could assess that the amount of acetaldehyde in sample WHO_2_ is totally combined with SO_2_ (50 mg of acetaldehyde combines with 70 mg of SO_2_) and considering the negligible levels of free sulfur dioxide after 11 months of aging, it is expected that further exposures to oxygen may lead to the appearance of free acetaldehyde.

In red wines, acetaldehyde concentration does not differ among the analyzed samples. This is probably due to the fact that, in presence of anthocyanins and higher concentration of flavanols, acetaldehyde is involved in a number of reactions with these phenolics during the aging. As discussed below, the most important reaction involving acetaldehyde, anthocyanins and flavanols is the formation of ethyl-bridged compounds [18,19] and ethyl-linked oligomers, which can further react with additional acetaldehyde, anthocyanins, and flavanols to generate a pyran ring, or other polymeric-type structures. Ultimately, these products can alter the wine sensory attributes [20] by affecting some key wine characteristics such as color, flavor and astringency.

### 2.2. Effect on Pigments and Chromatic Characters

**Figure 1:**
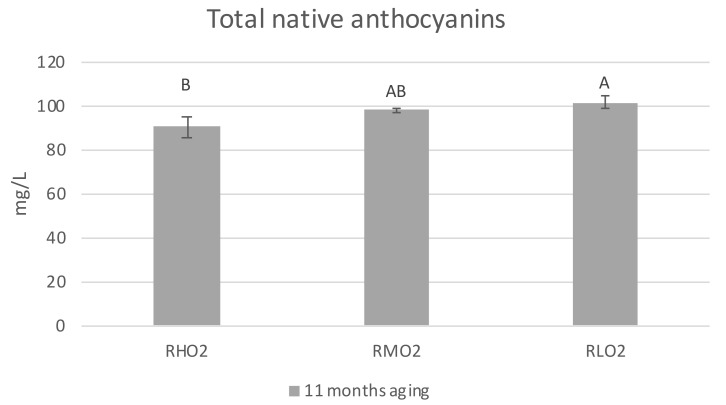
Total native anthocyanin. RHO_2_ (red high oxygen), RMO_2_ (red medium oxygen), RLO_2_ (red low oxygen). All the data are expressed as means ± standard deviation. Different letters indicate a statistically significant difference among treated wines. All the data are expressed as means ± standard deviation, (*p* < 0.05).

Data on the content of monomeric anthocyanins in treated red wines after 11 months of bottle aging showed (Table 3 and Supplementary Materials Figure S1) a loss of Malvidin-3-glucoside in the RHO_2_ sample with a higher concentration of oxygen than in RMO_2_ and RLO_2_. Consistently, differences in terms of total anthocyanins were detected among wines (Figure 1). Wine with a low concentration of oxygen at bottling showed a higher concentration of total native anthocyanins compared to the ones with higher concentrations of dissolved oxygen. The effect of the membrane contactor treatment on various pigment classes, determined by the Harbertson method, included an expected significant low concentration in SPP (short polymeric pigments) in samples RLO_2_ compared to the samples RHO_2_ and RMO_2_ that showed the highest increase of these important stable compounds (Table 4). LPP (long polymeric pigments) were not significantly different in all samples. Polymeric pigments (SPP and LPP) are defined as pigments resistant to bisulfite bleaching. They are formed by the reaction between anthocyanins and tannins during the wine aging [21], leading to a stabilization of the color over time. The main difference between these two classes of pigments is that, unlike SPP, LPP tend to precipitate with protein [22]. As red wine ages, a greater formation of LPP compared to SPP is usually observed. Thus, the changes detected for SPP and not for LPP can reflect an early oxidative state of Aglianico wines after 11 months of aging. The involvement of native anthocyanins in reactions yielding new polymeric pigments is consistent with the decrease of total native anthocyanins shown in (Table 4), and with similar effects observed in red wines during micro-oxygenation [23].

As wine ages and through different oxygen exposures, these polymeric pigments become of crucial importance for the wine color and small amounts of acetaldehyde can react with anthocyanins to produce new stable red pigments [19,24]. The fact that significant differences in terms of color intensity and hue were not detected (Table 5) could be probably due, as already observed for LPP, to the relatively short time of aging.

### 2.3. Effect on Red Wine Pigments: NMR and HR ESIMS Analyses

To the aim of understanding the molecular basis of the observed changes in the wines treated with different levels of oxygen, samples of RHO_2_, RMO_2_, and RLO_2_ were subjected to NMR-based analysis, as described in the Section 3. A careful inspection of the obtained ^1^H-NMR spectra of the three samples did not bring to light any discernible difference among the three compared wines (Supplementary Materials Figure S2). Hence, we decided to investigate the same wines by means of HR ESIMS given the intrinsic higher sensitivity of the technique when compared to NMR spectroscopy. The three wines were fractionated by HPLC/Vis by using a C-18 column. Three fractions were obtained for each wine: the first fraction was collected from 15 to 25 min (fraction 1), the second one from 25 to 30 min (fraction 2) and, finally, a third fraction (fraction 3) from 30 min through the end of the chromatographic run. Consistently with data reported in literature [25], in the first fraction non-acetylated anthocyanins were expected to be occurring, while potential pyranoanthocyanins were to be collected in the second fraction. The three obtained fractions (1–3) for each of the three analyzed wines (RHO_2_, RMO_2_ and RLO_2_) were all subjected to full scan HR ESIMS analysis in the positive ion mode. In fraction 1 of all wines, we detected an ion peak that was assigned to malvidin-3-*O*-glucoside (493.1332; Δ = −1.648; corresponding to C_23_H_25_O_12_^+^), whilst ion peaks related to the other common wine anthocyanins, when detected, presented errors above 10 ppm and were not considered reliable (Supplementary Materials Figure S3). In regard to fractions 2 and 3 of RMO_2_ and RLO_2_, they turned out to be basically superimposable with each other, while the mass spectra of fractions 2 and 3 of the RHO_2_ wine showed some interesting peculiarities. More specifically, in RHO_2_ fraction 2 an ion peak centered at m/z 517.1317 (Δ = −4.569; corresponding to C_25_H_25_O_12_^+^) was observed. This peak was attributed to Vitisin B (Supplementary Materials Figure S4) [26]. In the mass spectrum of fraction 3 of RHO_2_ two ion peaks at m/z 809.2294 (Δ = 0.827; corresponding to C_40_H_41_O_18_^+^) (Supplementary Materials Figure S5) and 1029.2871 (Δ = −0.065; corresponding to C_48_H_53_O_25_^+^) (Supplementary Materials Figure S6), respectively, were contained. These ion peaks were indicative of the occurrence of polymeric pigments. The peak at m/z 809 was attributed to ethylidene-bridged dimers constituted by one malvidin-3-*O*-glucoside unit and one (epi)catechin moiety [27], and the peak at m/z 1029 was assigned to the ethylidene-bridged dimer constituted by two malvidin-3-*O*-glucoside unit, of which one occurred in its flavylium form and the other one in its pseudobase form [28,29].

Vitisin B and ethylidene-bridged pigments (m/z 809 and 1029) are the result of the chemical reaction between acetaldehyde and anthocyanins or flavan-3-ols. Acetaldehyde can act either as a nucleophile at its alpha position or as an electrophile at the carbonyl functionality. The reaction between the nucleophile acetaldehyde and the electrophile C4 position of anthocyanins leads to the formation of Vitisin B, a quite stable compound classified as a pyranoanthocyanin. Conversely, when acetaldehyde acts as an electrophile by undergoing a nucleophilic attack by the C8, and to a lesser extent even by the C6 positions of either flavan-3-ols or anthocyanins, ethylidene-bridged dimers are formed. It is not surprising that we observed Vitisin B and red pigments only in the RHO_2_ wine, since such products, as discussed above, are formed by the reaction of acetaldehyde with anthocyanins and flavan-3-ols, and acetaldehyde is a molecule mainly resulting from the oxidative process undergone by wines over time by means of exposure to atmospheric oxygen. Hence, higher quantities of acetaldehyde are certainly present in RHO_2_ than in RMO_2_ and RLO_2_ wines that appear to have been protected from oxygen to a greater extent than RHO_2_.

### 2.4. Effect on VRF, BSA-Tannins and Total Phenols

Although soon after the membrane contactor treatment, the levels of total phenolics were similar among the treated wines, after 11 months of bottle aging, the amount of total phenols was higher in the sample with higher concentrations of oxygen at bottling, as shown in Table 6. This could be probably due to the role of oxygen played in the formation of phenolic compounds more reactive to iron and in variation of the molecular structure of monomeric and polymeric phenolic structures as already shown in wines undergoing different oxygen uptakes during the aging [6]. This is confirmed by the trend observed in Table 6 for BSA-reactive to tannins and flavans reactive to vanillin and in Table 4 for SPP, which showed a statistical difference after the 11 months of aging.

Tannin concentrations reactive to BSA-proteins were determined by the Habertson method in RHO_2_, RMO_2_ and RLO_2_. During the aging, an increase of the level of tannins reactive to BSA in RHO_2_ was observed, consistently with a possible polymerization of tannin structures [27]. Indeed, Harbertson [30] showed that the BSA precipitation increased as a function of the increasing degree of polymerization (or size) from trimers to octamers. As a consequence, every change in tannin composition and size can affect their capability of reacting with BSA. The oxidation of tannins causes the formation of intramolecular as well as intermolecular bonds between flavonoids. The latter cause the polymers to elongate and to become more reactive to salivary proteins [31]. These types of reactions can in fact modify the tannin structure and, thus, the hydrogen bonds and hydrophobic interactions with proteins [32].

As astringency is caused by the tannin-induced aggregation and the precipitation of salivary proteins [33], the increase of BSA-tannins in RHO_2_ suggests that the changes undergone by tannins by means of these reactions during the aging could contribute to modify the astringency perception.

Vanillin-reactive flavans (VRF) can instead provide further information related to the size of condensed tannins. In fact, vanillin reacts with the A ring of flavanols at either position 6 or 8 but also acetaldehyde reacts with the same positions of the A ring of flavanols. Therefore, a decrease of VRF may be regarded as an indirect measure of the oxidative polymerization of flavanols linked to reactions triggered by acetaldehyde and involving tannins and anthocyanins [34].

In our study, a slightly lower concentration of VRF was observed in the RMO_2_ sample. The lack of a clear trend as a function of the oxygen amount could be due to the fact that these molecules can also undergo hydrolytic cleavage in presence of oxygen by new molecular rearrangements with the formation of new intra and intermolecular bonds [6].

## 3. Materials and Methods

### 3.1. Wines

Two commercial red Aglianico and white Falanghina wines produced in Southern Italy by Cantina del Taburno winery were used. Details of the samples together with some base parameters are shown in Table 7. Base parameters were determined according to the OIV compendium of international methods of wine and must analysis (2007).

### 3.2. Wine Oxygen Management

An industrial system ISIOX (Tebaldi s.r.l) equipped with a gas-liquid membrane contactor (Liqui-Cel^®^, Transverse-flow, South Lakes Dr. Charlotte, NC, USA, cut off 50 g/mol) and a centrifugal pump in stainless steel was used. The membrane provides a fixed and well-determined interface for gas/liquid mass transfer without dispending one phase into another. The structure of the membrane contactor (hydrophobic hollow fiber) is made of polypropylene (PP) and offers a very large contact area (gas/liquid) of 20 m^2^.

The deoxygenation process consists of continuous cycles in which N_2_, vacuum or a combination of the first two processes (mix) circulating on one surface of polypropylene membrane is gradually enriched by oxygen deriving from wine circulating on the other side of the membrane. The driving force for the process is the difference in partial pressure of oxygen across the membrane. During the process, wine continuously circulates from a closed tank to the deoxygenation apparatus.

In order to achieve the required level of oxygen, the oxygen content in the wine was monitored through all the processes until the target level was reached.

The process control was carried out by monitoring O_2_ level in wines by using a PC incorporated with a very simple programming logic and specific sensors, which monitor the temperature and the oxygen content (luminescence system, Hach Lange, measurement range 0 to 20 mg/L O_2_, resolution: 0.01 mg/L O_2_, accuracy: 0–5 mg/L O_2_ ± 0.1). To control the inlet and outlet pressure, electronic pressure switches were used, as well as the pressure of the gas in the process and the level of any vacuum.

Different deoxygenation treatments were applied to wine in order to obtain different samples at different oxygen content. The amount of oxygen in white wine was respectively white High O_2_, WHO_2_ = 2.7 mg/L, white Medium O_2_, WMO_2_ = 1 mg/L, white Low O_2_, WLO_2_ = 0.25 mg/L.

The amount of oxygen in red wine was respectively red high O_2_, RHO_2_ = 1 mg/L, red Medium O_2_, RMO2 = 0.5 mg/L, red Low O_2_, RLO2 = 0.2 mg/L. Both sets of wines were filtered at 1 μm before being injected into the machine, with the purpose of avoiding fouling and wetting membrane phenomenon [35].

### 3.3. Samples Bottling and Aging

After the treatments, wines were bottled and all bottles were sealed using Nomacorc coextruded synthetic closures (Nomacorc SA, Thimister Clermont, Belgium) select green 100, which allow oxygen to pass through the cork in a controlled manner (0.4 mg O_2_ after 3 months, 0.7 mg O_2_ after 6 months, 1.2 mg O_2_ after 12 months and 1.1 mg O_2_ year after the first year of aging). Bottles were aged for 11 months at 15 °C.

### 3.4. Methods of Analysis

#### 3.4.1. High-Performance Liquid Chromatography Determination of Acetaldehyde

Analyses of acetaldehyde were performed by derivatizing experimental sample wine and HPLC.

The derivatization analysis was as follows: 100 μL of wine sample was dispensed into a vial, followed by the addition of 20 μL of freshly prepared 1.120 mg/L SO_2_ solution, 20 μL of 25% sulfuric acid (Carlo Erba reagent 96%), and 140 μL of 2 g/L 2,4-dinitrophenylhydrazine reagent. After mixing, the solution was allowed to react for 15 min at 65 °C and then promptly cooled to room temperature [36].

The HPLC used was a Shimadzu LC10 ADVP apparatus (Shimadzu Italy, Milan, Italy) equipped with a SCL-10AVP system controller, two LC-10ADVP pumps to create the needed solvent gradient, a SPD-M 10 AVP detector and an injection system full Rheodyne model 7725 (Rheodyne, Cotati, CA, USA). The separation was carried out on a Waters Spherisorb column (C 18, Silica particle substrate, ODS2 250 × 4.6 mm, 5 μm particles diameter, 80 Å pore size) equipped with a guard column. Optimum efficiency of separation was obtained using a flow rate of 0.75 mL/min, column temperature of 35 °C; mobile phase solvents were: (A) 0.5% formic acid (Sigma Aldrich ≥ 95%) in water milli-Q (Sigma Aldrich) and (B) acetonitrile (Sigma Aldrich ≥ 99,9%); gradient elution protocol was: 35% B to 60% B (t = 8 min), 60% B to 90% B (t = 13 min), 90% B to 95% B (t = 15 min, 2-min hold), 95% B to 35% B (t = 17 min, 4-min hold), total run time, 21 min, samples injections 50 µL and the detection was performed by monitoring the absorbance signals at 365 nm.

The calibration curves were made up by injecting 5 solutions (in triplicate) containing their respective standards covering the range of linearity 10–120mg/L and were characterized by a correlation coefficient (R^2^) > 0.976. Three analytical replicates were carried out for each experimental replicate.

#### 3.4.2. High-Performance Liquid Chromatography Analyses of Anthocyanins

Analyses of native anthocyanins were performed by a HPLC Shimadzu LC10 ADVP apparatus (Shimadzu Italy, Milan, Italy) equipped with a SCL-10AVP system controller, two LC-10ADVP pumps to create the needed solvent gradient, an SPD-M 10 AVP detector and an injection system full Rheodyne model 7725 (Rheodyne, Cotati, CA, USA). According to the method described in the OIV Compendium of International Methods of Analysis of Wine and Musts [37].

The HPLC solvents were the following: solvent A: water milli-Q (Sigma-Aldrich, Milan, Italy)/formic acid (Sigma-Aldrich ≥ 95%)/acetonitrile (SigmaAldrich ≥ 99.9%) (87:10:3) *v*/*v*; solvent B: water/formic acid/acetonitrile (40:10:50) *v*/*v*. The gradient was: zero-time conditions 94% A and 6% B; after 15 min, the pumps were adjusted to 70% A and 30% B, at 30 min to 50% A and 50%B, at 35 min to 40% A and 60% B, at 41 min, end of analysis, to 94% A and 6% B. A 5 min re-equilibration time was applied before the successive analysis as reported by [38]. The column used for the analyses was a waters spherisorb column (C 18, Silica particle substrate, ODS2 250 × 4.6 mm, 5 μm particles diameter, 80 Å pore size) with a precolumn was used. An amount of 50 µL of calibration standards or wine was injected onto the column. The absorbance signals at 520 nm were detected. Detector sensitivity was 0.01 Absorbance units full scale (AUFS). All the samples were filtered through 0.45 µm Durapore membrane filters (Millipore-Ireland) into glass vials and immediately injected into the HPLC system.

The calibration curve was obtained by injecting 5 solutions (in triplicate) containing increasing concentrations of malvidin-3-monoglucoside (Extrasynthese, Lyon, France). The calibration was characterized by a correlation coefficient (R^2^) = 0.996. The linearity range of the calibration curve was 2–200 mg/L. The precision of the method used was tested by six replicate analyses of a red wine sample containing 118.4 mg/L of total monomeric anthocyanins. The coefficient of variation was included between 1.1% (for malvidin 3-monoglucoside) and 9.1% (for malvidin 3-(6II-coumaroyl)-glucoside) and demonstrated the good reproducibility of the HPLC analysis. The monomeric anthocyanins concentrations were expressed as mg/L of malvidin-3-monoglucoside.

Fractionation of RLO_2_, RMO_2_ and RHO_2_ wines was performed according to the OIV method of analysis by using the same HPLC Shimadzu LC10 ADVP as reported above. Three analytical replicates were carried out for each experimental replicate.

#### 3.4.3. High Resolution Electrospray Ionization Mass Spectrometry (HR ESIMS) Analyses of Red Wines

The HPLC-separation of red wines conducted as described above afforded three fractions for each wine. The first fraction was collected from 15 to 25 min (fraction 1), the second one from 25 to 30 min (fraction 2) and the third fraction (fraction 3) from 30 min through the end of the chromatographic run (45 min). Each collected fraction was dried, solubilized in methanol and analyzed by HR-ESIMS in continuous flow injection in the positive ion mode. HR ESIMS experiments were performed on an Agilent 1260 Infinity II HPLC quaternary system coupled to a linear ion trap LTQ Orbitrap XL hybrid Fourier transform MS (FTMS) instrument equipped with an ESI ION MAX source (Thermo-Fisher). The following source settings were used (mass range *m*/*z* 100–2000): spray voltage 4.5 kV, capillary temperature 300 °C, capillary voltage 15 V, sheath gas 20 and auxiliary gas 21 (arbitrary units), tube lens voltage 140 V, and 25% collision energy. Calculation of elemental formulae was conducted by using Xcalibur software v 2.0.7 with a mass tolerance constrain of 5 ppm.

#### 3.4.4. NMR Experiments

An amount of 2 mL of each wine sample (RHO_2_, RMO_2_ and RLO_2_) was lyophilized and solubilized in 700 µL of CD_3_OD (δ_H_ 3.31; δ_C_ 49.0 ppm). NMR experiments were run on a Varian Unity Inova 700 spectrometer equipped with a 13C Enhanced HCN Cold Probe and by using a Shigemi 5 mm NMR tubes. The ^1^H-NMR standard Varian pulse sequence was employed.

#### 3.4.5. Standard Chemical Analyses and Spectrophotometric Measurement

According to “OIV Compendium of International Wine and Must Analysis of Wine and Musts Analysis 2007” [37], standard chemical analysis (alcohol strength calculated by volume, reducing sugar, total acidity, pH, volatile acidity, malic acidity and total dry matter) was measured.

Spectrophotometric analyses were performed by a Jenway 7305 spectrophotometer. Chromatic characteristics, color intensity and hue was determined according to OIV methods [37]. The color intensity was determined as the sum of abs 420 nm, abs 520 nm and abs 620 nm and hue as abs 420 nm/abs 520 nm ratio.

BSA-reactive tannins, short polymeric pigments (SPP), large polymeric pigments (LPP) and total phenols were determined by the Harbertson et al. assay [22]. Short polymeric pigments (SPP) and large polymeric pigments (LPP) were obtained by combining analysis of supernatant obtained after protein precipitation using bovine serum albumin BSA (Sigma-Aldrich) with the bisulphite bleaching of pigments in wine. BSA-tannin complex in the pellet was redissolved, added with ferric chloride and read at 510 nm. Total phenols were quantified by reading at 510 nm the sample as follows: 50 μL of wine was added to 825 μL of buffer C and read at the spectrophotometer as a blank solution. After the addition of 125 μL of ferric chloride, the sample was read again to quantify the amount of iron-reactive phenols.

Vanillin-reactive flavans (VRF) were determined according to Gambuti et al. [39]. Briefly, 750 μL of a solution of vanillin (4% in methanol) was added to 125 μL of diluted wine and, after 5 min, 375 μL of concentrated hydrochloric acid was added. After a 15-min incubation of the mixture at 20 °C, the absorbance was determined at 500 nm and read against a blank solution in which pure methanol was used instead of the solution of the solution of vanillin. Concentrations were calculated as (+)-catechin (mg/L).

#### 3.4.6. Statistical Analysis

Quantitative data were compared using Tukey’s least significant differences procedure, all the variance resulted homogeneous. When the variances were not homogeneous, data were analyzed using Kruskal–Wallis test. When results of the Kruskal–Wallis test were significant (*p* < 0.05), the significance of between-group differences was determined by the Bonferroni–Dunn test (5% significance level). These analyses were performed using XLSTAT (version 2013.6.04; Addinsoft, Paris, France). All data are means of three values.

## 4. Conclusions

Results obtained in this study confirmed that different contents of oxygen in bottled wine have an impact on wine aging and oxidation.

The oxygen management in the Aglianico wine by means of a polypropylene hollow fiber membrane contractor determined, after 11 months of aging, a lower content of free and total SO_2_ in sample with higher level of dissolved oxygen. The same behavior was observed in the Falanghina wine with an increase in terms of acetaldehyde in the sample with higher levels of oxygen.

In regard to phenolic compounds, a greater loss of total native anthocyanins was observed in red wines. Their content was higher in the wine with lower concentration of oxygen. However, the loss of total native anthocyanins did not affect the color parameters of wines, such as color intensity and tonality.

BSA-reactive tannins and vanillin-reactive flavans were lower in samples containing medium and low level of oxygen with respect to the samples with higher contents of oxygen. This is due to the fact that oxygen, by participating to oxidation reactions, concurs to the formation of polymeric pigments. As expected, in red wines with the highest content of dissolved oxygen after the membrane contactor treatment, Vitisin B, ethylidene-bridged dimers and acetaldehyde were more abundant in comparison to wines treated as to have a lower content of oxygen in pre-bottling phase.

In conclusion, the deoxygenation of wine by membrane contractor could be a suitable technique for wine industry to prevent all those oxidative phenomena that could change the final quality of red and white wines affecting the content of sulfur dioxide and acetaldehyde (especially in white wines). Nevertheless, oenologists have to consider that the decrease of oxygen content may affect the color stability in red wines.

## Figures and Tables

**Table 1:** Evolution of free sulfur dioxide and total sulfur dioxide after 11 months of aging of treated red Aglianico and white Falanghina wines.

**Aglianico**			
	**RHO** **_2_**	**RMO** **_2_**	**RLO** **_2_**
Free SO_2_	1.28 ± 0.00 B	1.60 ± 0.45 B	3.84 ± 0.00 A
Total SO_2_	33.92 ± 0.00 B	36.16 ± 0.45 AB	37.44 ± 1.36 A
**Falanghina**			
	**WHO** **_2_**	**WMO** **_2_**	**WLO** **_2_**
Free SO_2_	4.80 ± 0.45 B	7.36 ± 0.45 A	8.64 ± 0.45 A
Total SO_2_	67.2 ± 0.91 B	79.68 ± 0.45 A	78.08 ± 0.91 A

RHO_2_ (red high oxygen), RMO_2_ (red medium oxygen), RLO_2_ (red low oxygen), WHO_2_ (white high oxygen), WMO_2_ (white medium oxygen), WLO_2_ (white low oxygen). Different letters indicate a statistically significant difference among treated wines. All the data are expresses as means (mg/L) ± standard deviation, (*p* < 0.05).

**Table 2:** Evolution of acetaldehyde after 11 months of aging of treated Falanghina white wines.

Falanghina	
Acetaldehyde (mg/L)	
WHO_2_	45.85 ± 0.11 A
WMO_2_	44.82 ± 0.43 B
WLO_2_	44.60 ± 0.15 B

WHO_2_ (white high oxygen), WMO_2_ (white medium oxygen), WLO_2_ (white low oxygen). All the data are expressed as means (mg/L) ± standard deviation. Different letters indicate a statistically significant difference among treated wines. All the data are expresses as means ± standard deviation, (*p* < 0.05).

**Table 3:** Native anthocyanins.

Aglianico			
	RHO_2_	RMO_2_	RLO_2_
Delf-3-gl	9.62 ± 0.02 A	9.98 ± 0.28 A	10.26 ± 0.40 A
Petu-3-gl	9.36 ± 4.47 A	13.40 ± 0.34 A	14.15 ± 0.47 A
Peon-3-gl	2.43 ± 0.26 A	2.66 ± 0.25 A	2.86 ± 0.22 A
Malv3-gl	56.94 ± 0.18 B	59.08 ± 0.46 AB	60.64 ± 1.54 A
Mal-3-Acgl	8.74 ± 0.05 A	9.29 ± 0.07 A	9.47 ± 0.38 A
Mal-3-Cumgl	2.44 ± 0.11 A	2.58 ± 0.20 A	3.08 ± 0.48 A

RHO_2_ (red high oxygen), RMO_2_ (red medium oxygen), RLO_2_ (red low oxygen). Delf-3-gl (Delphinidin-3-glucoside), Cyan-3-gl (Cyanidin-3-glucoside), Petu-3-gl (Petunidin-3-glucoside), Peon-3-gl (Peonidin-3-glucoside), Malv-3-gl (Malvidin-3-glucoside), Mal-3-Acgl (Malvidin-3-acetyglucoside), Mal-3-Cumgl (Malvidin-3-coumarylglucoside). All the data are expressed as means (mg/L) ± standard deviation. Different letters indicate a statistically significant difference among treated wines. All the data are expresses as means ± standard deviation, (*p* < 0.05).

**Table 4:** Polymeric pigments (SPP) Short polymeric pigments and (LPP) Long polymeric pigments.

Aglianico			
	RHO_2_	RMO_2_	RLO_2_
SPP	0.68 ± 0.02 A	0.65 ± 0.01 AB	0.64 ± 0.00 B
LPP	0.52 ± 0.03 A	0.52 ± 0.01 A	0.50 ± 0.02 A

RHO_2_ (red high oxygen), RMO_2_ (red medium oxygen), RLO_2_ (red low oxygen). All the data are expressed as means (Abs Units) ± standard deviation. Different letters indicate a statistically significant difference among treated wines. All the data are expresses as means ± standard deviation, (*p* < 0.05).

**Table 5:** Chromatic Characteristics.

Aglianico		
	Color Intensity	Hue
RHO_2_	8.09 ± 0.14 A	0.73 ± 0.00 A
RMO_2_	7.83 ± 0.16 A	0.73 ± 0.01 A
RLO_2_	7.84 ± 0.27 A	0.74 ± 0.01 A

RHO_2_ (red high oxygen), RMO_2_ (red medium oxygen), RLO_2_ (red low oxygen). All the data are expressed as means (Abs Units) ± standard deviation. Different letters indicate a statistically significant difference among treated wines. All the data are expresses as means ± standard deviation, (*p* < 0.05).

**Table 6:** Total phenols, flavans reactive to vanillin and tannins reactive to BSA.

Aglianico			
	RHO_2_	RMO_2_	RLO_2_
BSA Reactive Tannins	84.43 ± 19.14 A	41.51 ± 11.98 B	37.74 ± 26.39 B
FRV (mg/L)	1064.84 ± 66.66 A	931.69 ± 52.26 B	1001.21 ± 3.33 A
Total Phenols (mg/L)	534.85 ± 27.81 A	494.28 ± 6.75 B	474.79 ± 4.75 C

RHO_2_ (red high oxygen), RMO_2_ (red medium oxygen), RLO_2_ (red low oxygen). All the data are expressed as means ± standard deviation. Different letters indicate a statistically significant difference among treated wines. All the data are expresses as means (mg/L) ± standard deviation, (*p* < 0.05).

**Table 7:** Wine chemical parameters.

	Falanghina	Aglianico
Alcohol (*v*/*v*%)	13.30	13.85
Sugars (g/L)	3.03	1.14
pH	3.21	3.45
Total acidity (g/L)	5.68	5.44
Volative acidity (g/L)	0.21	0.32
Malic acid (g/L)	1.78	0.75
Dry extract (g/L)	21.57	27.82

## Data Availability

Not applicable.

## Supplementary Materials

The following are available online. Figure S1: Enlargements of ^1^H-NMR spectra of wines RHO_2_, RMO_2_ and RLO_2_ registered in CD_3_OD. Figure S2: Structure of malvidin 3-*O*-glucoside and relative HR ESIMS in the positive ion mode. Figure S3: Structure of Vitisin B and relative HR ESIMS in the positive ion mode. Figure S4: Structure of ethylidene-bridged dimer constituted by one unit of malvidin 3-*O*-glucoside (bottom) and one unit of (epi)catechin (top) with the relative HR ESIMS in the positive ion mode. Figure S5: Structure of ethylidene-bridged dimer constituted by two units of malvidin 3-*O*-glucoside, of which the one at the bottom is in its flavylium form and the one on the top in its pseudobase form, along with the relative HR ESIMS in positive ion mode.

## References

[1] Waterhouse A.L., Laurie V.F. (2006). Oxidation of wine phenolics: A critical evaluation and hypotheses. Am. J. Enol. Vitic..

[2] Danilewicz J.C. (2011). Mechanism of autoxidation of polyphenols and participation of sulfite in wine: Key role of iron. Am. J. Enol. Vitic..

[3] Nikolantonaki M., Waterhouse A.L. (2012). A method to quantify quinone reaction rates with wine relevant nucleophiles: A key to the understanding of oxidative loss of varietal thiols. J. Agric. Food Chem..

[4] Elias R.J., Andersen M.L., Skibsted L.H., Waterhouse A.L. (2009). Identification of free radical intermediates in oxidized wine using electron paramagnetic resonance spin trapping. J. Agric. Food Chem..

[5] Oliveira C.M., Ferreira A.C.S., De Freitas V., Silva A.M. (2011). Oxidation mechanisms occurring in wines. Food Res. Int..

[6] Mouls L., Fulcrand H. (2012). UPLC-ESI-MS study of the oxidation markers released from tannin depolymerization: Toward a better characterization of the tannin evolution over food and beverage processing. J. Mass Spectrom..

[7] Waterhouse A.L. (2002). Wine phenolics. Ann. N. Y. Acad. Sci..

[8] Rosenfeld E., Schaeffer J., Beauvoit B., Salmon J.M. (2004). Isolation and properties of promitochondria from anaerobic stationary-phase yeast cells. Antonie Van Leeuwenhoek.

[9] Jarauta I., Cacho J., Ferreira V. (2005). Concurrent phenomena contributing to the formation of the aroma of wine during aging in oak wood: An analytical study. J. Agric. Food Chem..

[10] Singleton V.L., Trousdale E., Zaya J. (1979). Oxidation of wines. 1. Young white wines periodically exposed to air. Am. J. Enol. Vitic..

[11] Gabelman A., Hwang S.T. (1999). Hollow fiber membrane contactors. J. Membr. Sci..

[12] Gambuti A., Rinaldi A., Lisanti M.T., Pessina R., Moio L. (2011). Partial dealcoholisation of red wines by membrane contactor technique: Influence on colour, phenolic compounds and saliva precipitation index. Eur. Food Res. Technol..

[13] Blank A., Vidal J.C. (2012). Gas management by membrane contactor: Ester and higher alcohol losses, and comparison with porous injector. Bull. l’OIV.

[14] Schonenberger P., Baumann I., Jaquerod A., Ducruet J. (2014). Membrane Contactor: A Nondispersive and Precise Method to Control CO2 and O2 Concentrations in Wine. Am. J. Enol. Vitic..

[15] Waterhouse A.L., Sacks G.L., Jeffery D.W. (2016). Understanding Wine Chemistry.

[16] Jenkins T.W., Howe P.A., Sacks G.L., Waterhouse A.L. (2020). Determination of Molecular and “Truly” Free Sulfur Dioxide in Wine: A Comparison of Headspace and Conventional Methods. Am. J. Enol. Vitic..

[17] Bonaldo F., Guella G., Mattivi F., Catorci D., Arapitsas P. (2020). Kinetic investigations of sulfite addition to flavanols. Sci. Rep..

[18] Schmidtke L.M., Clark A.C., Scollary G.R. (2011). Micro-oxygenation of red wine: Techniques, applications, and outcomes. Crit. Rev. Food Sci. Nutr..

[19] Timberlake C.F., Bridle P. (1976). Interactions between anthocyanins, phenolic compounds, and acetaldehyde and their significance in red wines. Am. J. Enol. Vitic..

[20] He F., Liang N.N., Mu L., Pan Q.H., Wang J., Reeves M.J., Duan C.Q. (2012). Anthocyanins and their variation in red wines I. Monomeric anthocyanins and their color expression. Molecules.

[21] Fulcrand H., Dueñas M., Salas E., Cheynier V. (2006). Phenolic reactions during winemaking and aging. Am. J. Enol. Vitic..

[22] Harbertson J.F., Picciotto E.A., Adams D.O. (2003). Measurement of polymeric pigments in grape berry extract sand wines using a protein precipitation assay combined with bisulfite bleaching. Am. J. Enol. Vitic..

[23] Picariello L., Rinaldi A., Forino M., Errichiello F., Moio L., Gambuti A. (2020). Effect of Different Enological Tannins on Oxygen Consumption, Phenolic Compounds, Color and Astringency Evolution of Aglianico Wine. Molecules.

[24] Fulcrand H., dos Santos P.J.C., Sarni-Manchado P., Cheynier V., Favre-Bonvin J. (1996). Structure of new anthocyanin-derived wine pigments. J. Chem. Soc. Perkin Trans..

[25] Rentzsch M., Schwarz M., Winterhalter P., Blanco-Vega D., Hermosín-Gutiérrez I. (2010). Survey on the content of vitisin A and hydroxyphenyl-pyranoanthocyanins in Tempranillo wines. Food Chem..

[26] Bakker J., Timberlake C.F. (1997). Isolation, identification, and characterization of new color-stable anthocyanins occurring in some red wines. J. Agric. Food Chem..

[27] Es-Safi N.E., Fulcrand H., Cheynier V., Moutounet M. (1999). Studies on the acetaldehyde-induced condensation of (−)-epicatechin and malvidin 3-*O*-glucoside in a model solution system. J. Agric. Food Chem..

[28] Atanasova V., Fulcrand H., Le Guernevé C., Cheynier V., Moutounet M. (2002). Structure of a new dimeric acetaldehyde malvidin 3-glucoside condensation product. Tetrahedron Lett..

[29] Forino M., Picariello L., Lopatriello A., Moio L., Gambuti A. (2020). New insights into the chemical bases of wine color evolution and stability: The key role of acetaldehyde. Eur. Food Res. Tech..

[30] Harbertson J.F., Mireles M., Yu Y. (2014). Improvement of BSA tannin precipitation assay by reformulation of resuspension buffer. Am. J. Enol. Vitic..

[31] Poncet-Legrand C., Cabane B., Bautista-Ortín A.B., Carrillo S., Fulcrand H., Perez J., Vernhet A. (2010). Tannin oxidation: Intra-versus intermolecular reactions. Biomacromolecules.

[32] Watrelot A.A., Day M.P., Schulkin A., Falconer R.J., Smith P., Waterhouse A.L., Bindon K.A. (2019). Oxygen exposure during red wine fermentation modifies tannin reactivity with poly-L-proline. Food Chem..

[33] Soares S., Sousa A., Mateus N., de Freitas V. (2012). Effect of condensed tannins addition on the astringency of red wines. Chem. Sens..

[34] Gambuti A., Rinaldi A., Ugliano M., Moio L. (2012). Evolution of phenolic compounds and astringency during aging of red wine: Effect of oxygen exposure before and after bottling. J. Agric. Food Chem..

[35] Breniaux M., Zeng L., Bayrounat F., Ghidossi R. (2019). Gas transfer management by membrane contactors in an oenological context: Influence of operating parameters and membrane materials. Sep. Purif. Technol..

[36] Han G., Wang H., Webb M.R., Waterhouse A.L. (2015). A rapid, one step preparation for measuring selected free plus SO2- bound wine carbonyls by HPLC-DAD/MS. Talanta.

[37] OIV (2017). Compendium of International Methods of Wine and must Analysis.

[38] Picariello L., Gambuti A., Picariello B., Moio L. (2017). Evolution of pigments, tannins and acetaldehyde during forced oxidation of red wine: Effect of tannins addition. LWT Food Sci. Technol..

[39] Gambuti A., Han G., Peterson A.L., Waterhouse A.L. (2015). Sulfur dioxide and glutathione alter the outcome of microoxygenation. Am. J. Enol. Vitic..

