# Supplementary material for: The Management of Dissolved Oxygen by a Polypropylene Hollow Fiber Membrane Contactor Affects Wine Aging
Source: Molecules. 2021 Jun 11;26(12):3593. doi: 10.3390/molecules26123593 (PMC8231238; doi:10.3390/molecules26123593)

Supplementary Material Figure S1

Comparison among the HPLC chromatograms of anthocyanins of wines RHO_2, RMO_2 and RLO_2.

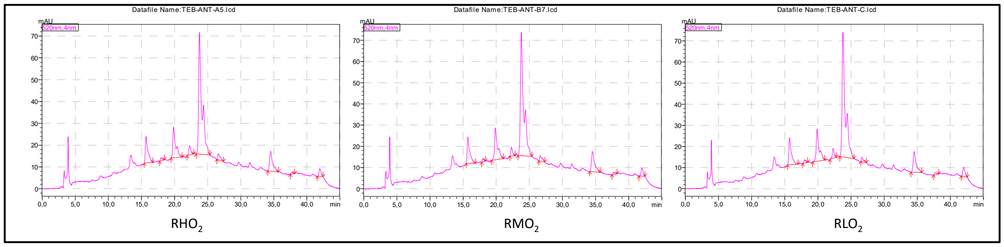

Supplementary Material Figure S2

Enlargements of 1H-NMR spectra of wines RHO2 (bottom), RMO2 (center) and RLO2 (top) registered in CD3OD. The shown spectral regions contain typical resonances of wine polyphenols.

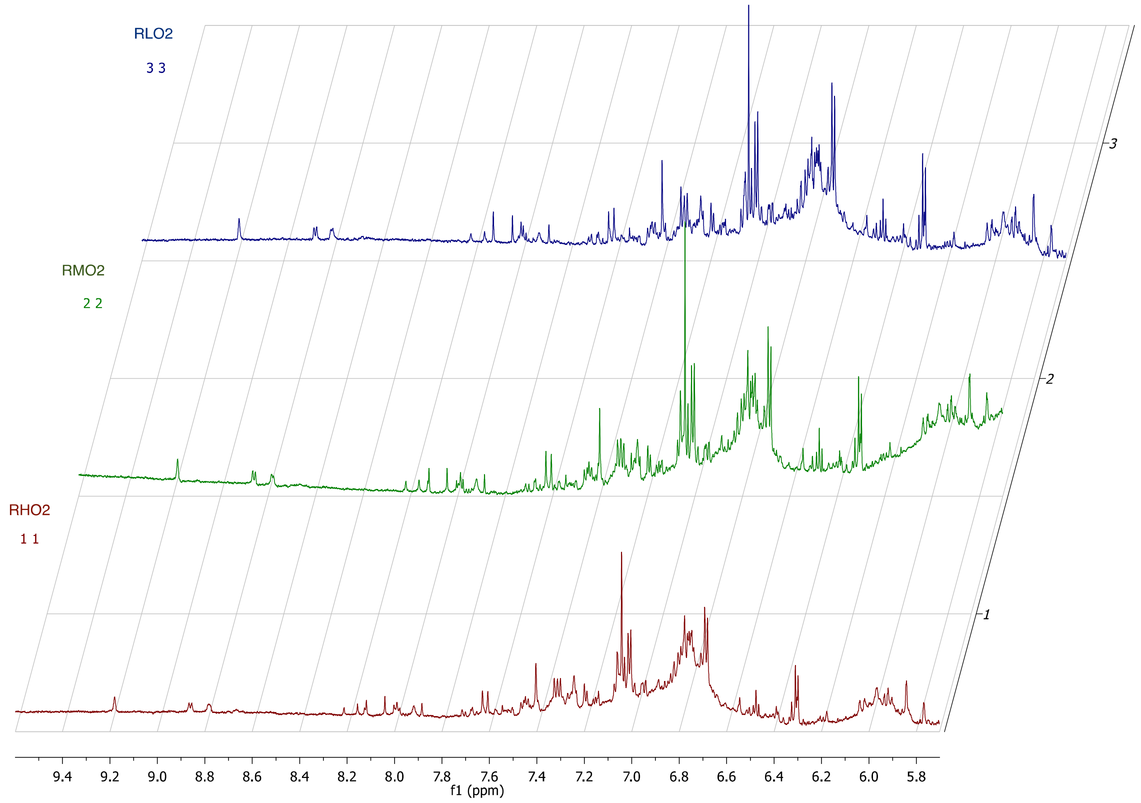

Supplementary Material Figure S3

Structure of malvidin 3-O-glucoside and relative HR ESIMS (positive ion mode) spectrum containing the relative ion peak at m/z 493

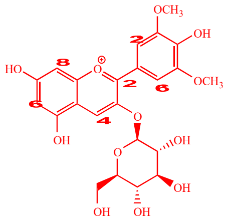

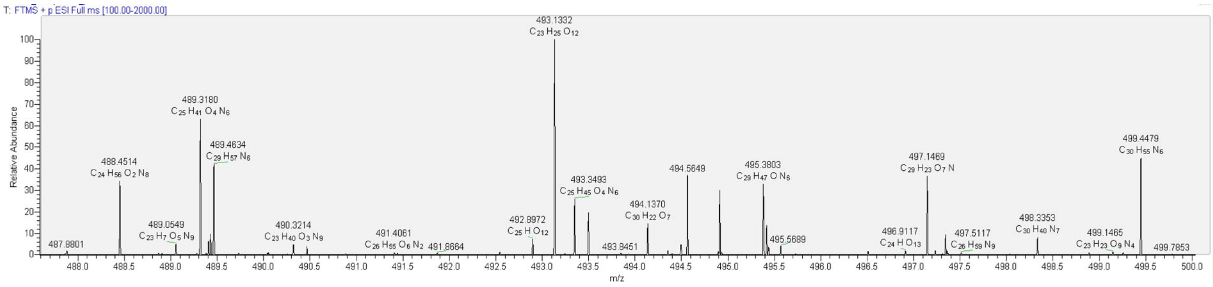

Supplementary Material Figure S4

Structure of Vitisin B and relative HR ESIMS (positive ion mode) spectrum containing the relative ion peak at m/z 517

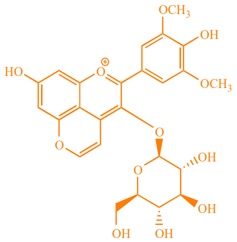

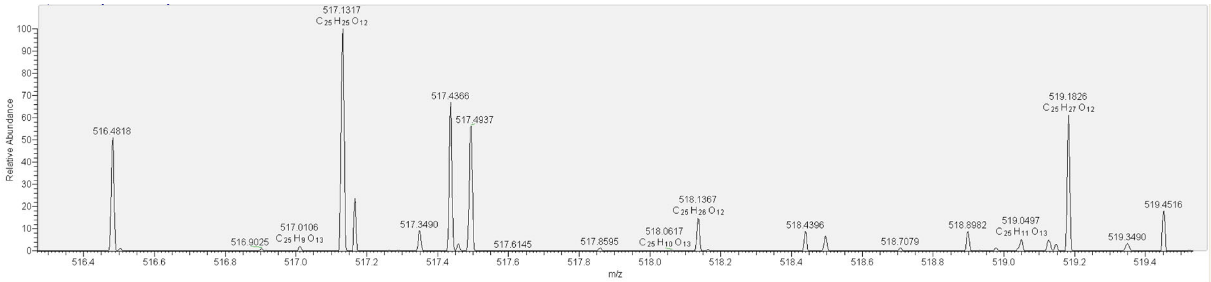

Supplementary Material Figure S5

Structure of ethylidene bridged dimer constituted by one unit of malvidin 3-O-glucoside (bottom) and one unit of (epi)catechin (top) with the relative HR ESIMS (positive ion mode) spectrum containing the ion peak at m/z 809. A possible ethylidene bridge connecting 8 and 6 positions of either catechin or malvidin cannot be ruled out on the basis of MS data.

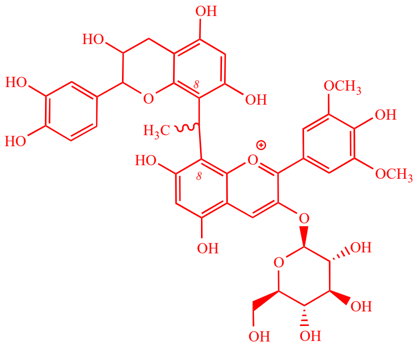

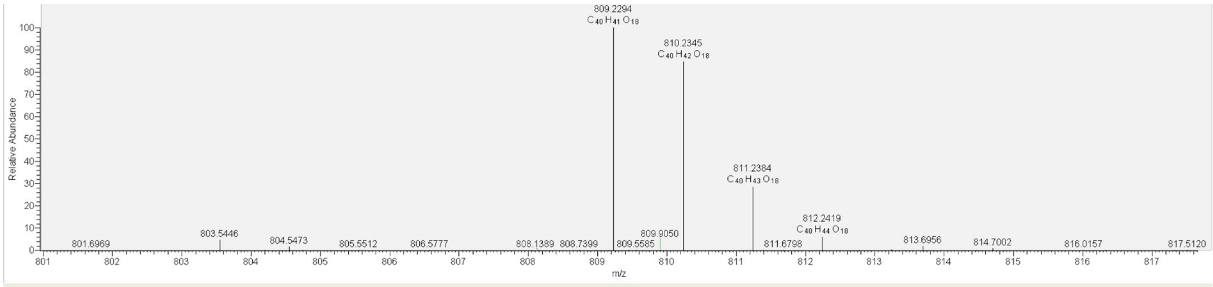

Supplementary Material Figure S6

Structure of ethylidene bridged dimer constituted by two units of malvidin 3-*O*-glucoside, of which the one at the bottom is in its flavylium form and the one on the top in its pseudobase form, along with the relative HR ESIMS (positive ion mode) spectrum containing the ion peak at *m/z* 1029. A possible ethylidene bridge connecting 8 and 6 positions of either unit cannot be ruled out on the basis of MS data.

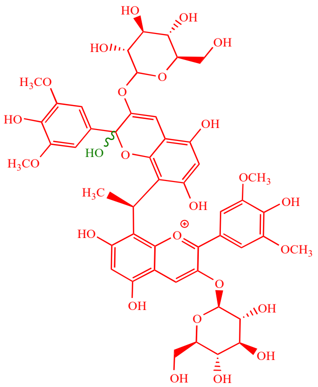

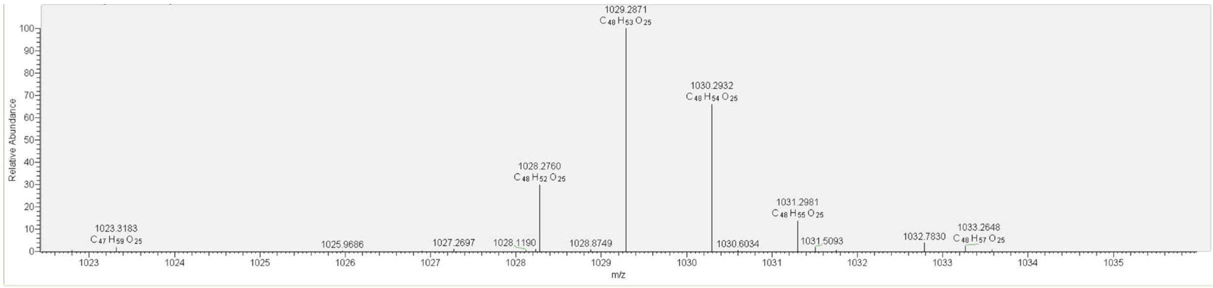

Supplement: Supplementary file 1 [file molecules-26-03593-s001.zip › molecules-1229282-supplementary.pdf]
